# Causal discovery in high-dimensional, multicollinear datasets

**DOI:** 10.3389/fepid.2022.899655

**Published:** 2022-09-13

**Authors:** Minxue Jia, Daniel Y. Yuan, Tyler C. Lovelace, Mengying Hu, Panayiotis V. Benos

**Affiliations:** ^1^Department of Computational and Systems Biology, University of Pittsburgh School of Medicine, Pittsburgh, PA, United States; ^2^Joint Carnegie Mellon - University of Pittsburgh Computational Biology PhD Program, Pittsburgh, PA, United States; ^3^Department of Epidemiology, University of Florida, Gainesville, FL, United States

**Keywords:** causal discovery, dimensionality reduction, latent factors, collinearity, empirical bayes matrix factorization

## Abstract

As the cost of high-throughput genomic sequencing technology declines, its application in clinical research becomes increasingly popular. The collected datasets often contain tens or hundreds of thousands of biological features that need to be mined to extract meaningful information. One area of particular interest is discovering underlying causal mechanisms of disease outcomes. Over the past few decades, causal discovery algorithms have been developed and expanded to infer such relationships. However, these algorithms suffer from the curse of dimensionality and multicollinearity. A recently introduced, non-orthogonal, general empirical Bayes approach to matrix factorization has been demonstrated to successfully infer latent factors with interpretable structures from observed variables. We hypothesize that applying this strategy to causal discovery algorithms can solve both the high dimensionality and collinearity problems, inherent to most biomedical datasets. We evaluate this strategy on simulated data and apply it to two real-world datasets. In a breast cancer dataset, we identified important survival-associated latent factors and biologically meaningful enriched pathways within factors related to important clinical features. In a SARS-CoV-2 dataset, we were able to predict whether a patient (1) had COVID-19 and (2) would enter the ICU. Furthermore, we were able to associate factors with known COVID-19 related biological pathways.

## Introduction

Technological advances have allowed the cost-effective collection of high-throughput data in unprecedented volume and rate. Such data can be used to uncover biological mechanisms of disease and develop predictors of disease status and progression (1, 2), response to drugs (3, 4) or define disease subtypes (5). However, such high-throughput datasets, with thousands to millions of features present two major problems to most analytical methods: high dimensionality, which creates a complexity problem, and high collinearity of the variables due to the underlying biological structure. For example, genes regulated by the same transcription factors (TFs) are usually co-expressed, genetic variants (single nucleotide polymorphisms—SNPs) in close proximity co-segregate, and expression of microRNAs correlates with their target genes.

The analytical questions on such data are usually two-fold: (1) mechanistic and (2) predictive. In the first category, the objective is to understand the mechanism that causes a biological phenomenon or a clinical outcome. The focus here is on uncovering the complex relations between features in the dataset. For example, how one gene affects the expression of another gene, how smoking, age, or sex affects gene expression, or whether a SNP contributes to drug response or clinical outcomes. The second question is about our ability to predict an outcome. Causal learning methods are emerging as a flexible tool for addressing both these types of questions (6, 7). For example, directed graphs learned from such observational data can be used to infer regulatory interactions between genes (5, 8) and the Markov blanket of an outcome can be used to build an efficient predictor of it (9, 10). However, causal learning methods also suffer from the curse of dimensionality and feature collinearity, which limit their applicability to high-throughput omics datasets.

To cope with these problems, supervised (11, 12) and unsupervised (13) methods for inferring latent feature spaces have been developed and used for identifying regulatory modules (14, 15) and for clustering or prediction (13, 16). Although much of this work has been applied to gene expression, it has also seen success in identifying biologically meaningful signals in DNA methylation data (17). In causal modeling literature, there are algorithms that can learn causal graphs in the presence of latent confounders (18–21), but they usually do not model the observed features as the result of those latent variables. However, it is expected that combining dimensionality reduction methods with causal learning methods will solve both problems that hinder causal discovery in modern biomedical datasets. Recently, a new method (CausER) (22) has been introduced, which first uses a form of soft clustering to infer latent factors that can explain all observed variables; and then uses causal discovery on the latent factors to build predictors of clinical outcomes.

**Figure 1:**
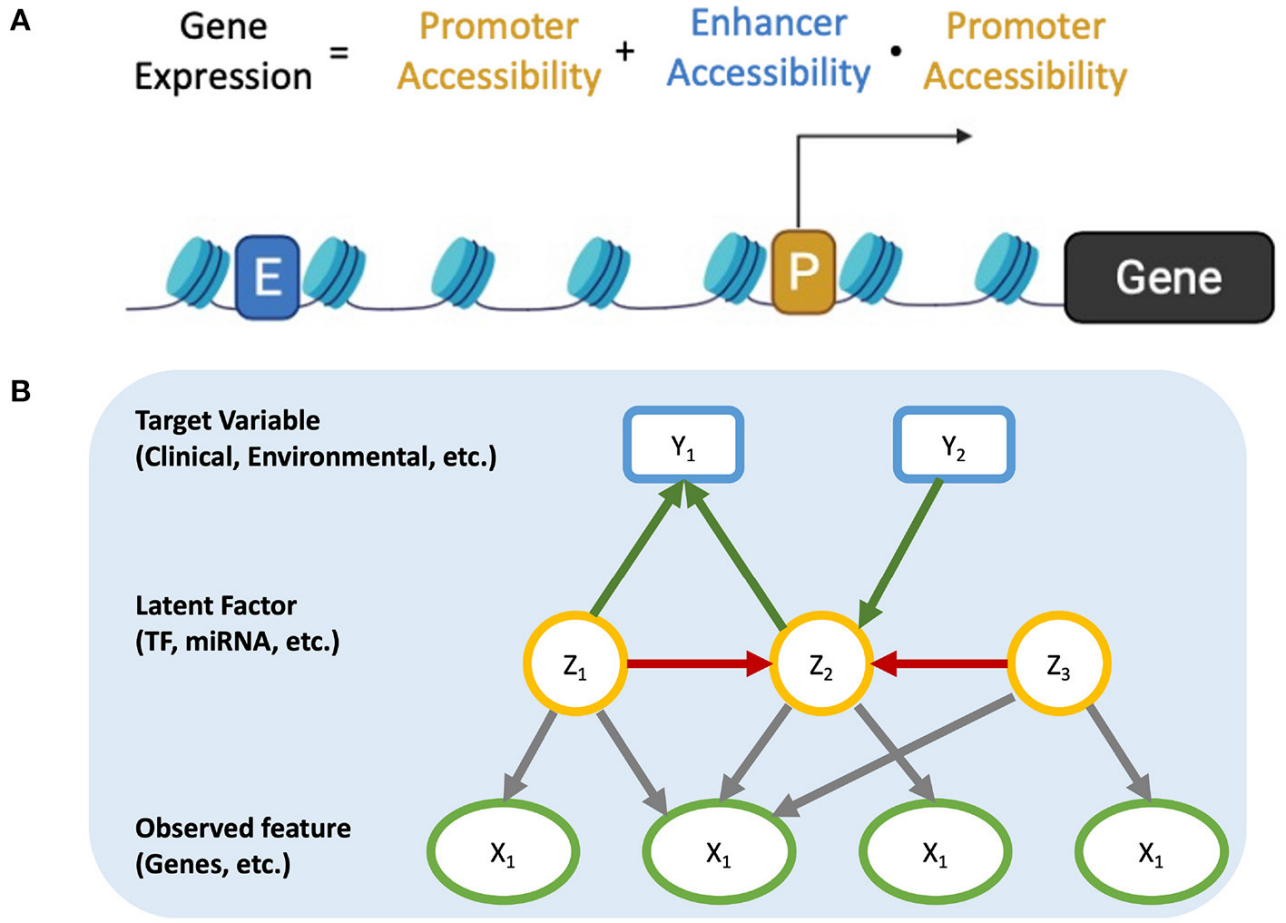
**(A)** Regulation of gene expression. **(B)** Causal Graph including relationship among observed features, latent factors, and target features.

Gene (mRNA) expression is regulated by proteins (transcription factors) that bind to DNA cis-regulatory modules of the genes. However, the protein levels of TFs are typically not measured in the same set up as the mRNA expression. In addition, the concordance between mRNA and protein levels can be as low as 25% (23, 24), and other factors, like chromatin accessibility and the presence of enhancers, might affect expression of a given gene. Figure 1A represents a simplified model on how accessible and active promoter and enhancer regions can regulate the transcriptional program of cells by binding to TFs. Promoters regulate the transcription of the downstream gene, while enhancers can loop over long-range genomic regions to interact with distal promoters. Thus, the expression of genes is mainly controlled by the accessibility of active enhancers and promoters to TF. We propose that this regulation model also suggests a possible solution.

Suppose we have a system where the observed variables are independent conditioned on all latent features. If we can extract latent features from the raw features, we can build a causal model between latent features, where there will be no direct interactions within the raw variables. Any causal associations found among latent features can then examined further by looking at the raw variables that comprise them. Such is the gene expression model depicted in Figure 1B. Gene-gene interactions do not happen at the mRNA level (the measured property) and there are multiple non-measured steps that are involved (translation, post-translational modifications, localization, etc.). These unmeasured events can be approximately represented by latent factors. In this scenario, the latent factors can capture TF activity (protein synthesis rate, post-translational modifications, localization, etc.) as well as genetic and epigenetic factors (such as chromosome structure, microRNAs, etc.), which also affect gene expression in a collective way (25–27).

In our paper, we introduce a new framework that combines a recently published approach for non-orthogonal dimensionality reduction (empirical Bayes approach to matrix factorization—EBMF) (28) with causal discovery to concurrently address both high dimensionality and collinearity issues. We demonstrate the utility of this framework by making useful predictions on two biomedical problems.

## Methods

### High-dimensional CausalMGM workflow

Biomarkers or features identified from high-dimensional biological data through regression or statistical tests are informative for prediction but the observed variables (mRNA expression) may not be directly (causally) linked to disease driver mechanisms or events. We propose a new framework that can identify latent factors causally linked to clinical features without use of prior knowledge. Our proposed workflow is composed of two parts. First, we learn the latent factors from the observed variables. Second, we learn the causal associations among latent factors and between latents and the target features (e.g., outcomes) (Figure 1B). For the first part, we selected EBMF due to its ability to learn non-orthogonal latent factors that are identifiable up to scale transformations. Additionally, Wang et al. demonstrated the ability of EBMF to recover sparse, biologically meaningful factors in gene expression data (28). For the second part, we use our CausalMGM framework, which has been shown to have superior performance on mixed data types (2, 29).

### Empirical bayes matrix factorization

EBMF is a recently introduced non-orthogonal, unsupervised method for dimensionality reduction. The *K*-factor EBMF model is defined in Equation (1) where *Y* is the *n* × *p* data matrix, **l**_*k*_ is an *n*-vector, **f**_*k*_ is a *p*-vector, G are pre-specified families of distributions, and *g* are unknown “prior” distributions, *E* is an *n*-specified families of distributions, and *p* matrix of independent error terms, and **τ** is an unknown *n*-specified families of distributions, and *p* matrix of precisions in some space T,


(1) $$\begin{array}{l} \;Y=\sum_{k=1}^{K}l_{k}f_{k}^{T}+E\\ l_{kl},\ldots ,l_{kn}\sim^{iid}g_{l_{k}},g_{l_{k}}\in G_{l}\\ f_{kl},\ldots ,f_{kp}\sim^{iid}g_{f_{k}},g_{f_{k}}\in {ℱ}_{f}\\ \;E_{ij}\sim N(0,1/\tau_{ij})with\tau :=(\tau_{ij})\in T \end{array}$$

Wang et al. (28) implemented two main algorithms for fitting the k-factor EBMF model: (1) greedy and (2) backfitting. The greedy approach starts by optimizing the first factor, then adding second factor and optimizing that, and so on one factor at a time. The backfitting approach uses the estimates of all factors to refine the estimate of one factor (30). Wang et al. also state that empirical Bayes approaches to matrix factorization can automatically select the number of factors *K* (31) because, if *K* is set sufficiently large, some loading/factor combinations will be optimized to 0. They also show that, in their EBMF model, each loading and factor is identifiable to a multiplicative constant (given that G is a scale family). This can also be dealt with by normalizing factor estimates.

### CausalMGM

#### PC and FCI

One of the most popular constraint-based causal discovery algorithms is the PC algorithm (32). One of the main assumptions of the PC algorithm is that the ground truth graph can be represented by a Directed Acyclic Graph (DAG). The PC algorithm starts with a fully connected undirected graph. It then tests each pair of variables for independence conditioned on the empty set and removes all those edges found to be independent. Then, the procedure is repeated by testing for conditional independence of each edge given every single neighborhood variable, every pair of neighborhood variables, etc. until a predetermined size. After every conditionally independent edge has been identified and removed, the remaining edges are oriented. The orientation has the following steps. First, if node Z is adjacent to nodes X and Y, and X and Y are independent conditioned on a set that does not include Z, then this is a collider (X → Z←Y). Next, the remaining edges are oriented to avoid additional colliders. Finally, any edges that are not in a collider, nor would result in a collider, are oriented to avoid cycles.

In the original PC algorithm, edges would be removed as soon as a conditional independence is found between two nodes. However, this meant that the output graph would be dependent on the order in which the edges were tested. PC-stable (33) corrects for this by performing all edge removals concurrently at the end of each depth (subset size) test. PC−Max further improves on this idea by picking the conditioning set with the highest *p*-value so that there are no ambiguities for directionality. In addition, PC−Max is parallelized for scalability (34).

The Fast Causal Inference (FCI) algorithm (32) is a modification of PC that has been explicitly designed for causal discovery in the presence of latent confounders. The FCI algorithm is composed of three parts: (1) determine which edges to remove from the complete undirected graph (2) identify collider and orient edges (3) re-orient edges for ancestor relations. The output of FCI is defined as partial ancestral graph (PAG) including four types of edge:

- *A*→*B* if and only if A is an ancestor of B in the I-equivalence class
- *A*↔*B* if and only if A and B are not ancestors of each other in the I-equivalence class
- A *o*−> B if and only if B is not an ancestor of A in the I-equivalence class
- A *o*−*o* B places no restriction on ancestor relation.

#### FGES

The Fast Greedy Equivalence Search (FGES) algorithm (35) is one of the most popular score-based causal discovery algorithm, which infers causal structure through maximizing a likelihood score instead of conditional independence tests. Recently, FGES has been extented to mixed-type data *via* Degenerate Gaussian score (36) where the likelihood is calculated by modeling continuous random variable as Gaussian distributions, and each k-category discrete random variable as a latent (k – 1) dimensional Gaussian distributions. The output of FGES is pattern, which contains directed edge (→) representing direct causation and undirected edge (−), where its causal direction cannot be determined.

#### Mixed graphical models

The main limitation of constraint-based causal algorithms (like PC−Max) is that the runtime in dense or scale-free graphs is a high-order polynomial based on the maximum degree of the graph. One way to improve upon this is to start the search from a sparse undirected skeleton that includes all adjacencies in the true data generating DAG. This reduces a global high-order polynomial problem to a local lower-order polynomial problem. Mixed graphical model (MGM) is one strategy that allows for the efficient learning of the moralized graph (which is a superset of the true causal graph plus the edges for the shielded colliders) (37, 38). MGM can learn a undirected graph skeleton and avoid a large number of false positive edges (2).

## Experiments

### Comparison on synthetic datasets

#### Synthetic datasets

Clinical datasets often contain a mixture of continuous and discrete variables. When multi-omics data is included, these datasets become high-dimensional and collinear. To simulate similar datasets, we used both Lee and Hastie (LH) (37) and Conditional Gaussian (CG) (39) models for data generation. Conditional Gaussian models generate data from a Gaussian mixture where each Gaussian component exists for a particular combination of discrete variables, while Lee & Hastie models generate continuous variables from the joint distribution of the continuous variables conditioned on the discrete variables following a multivariate Gaussian with common covariance.

The TETRAD software (40, 41) contains LH and CG simulation models. We generated 10,000 samples for 50 continuous variables (representing factors *Z*) and 25 categorical variables (analogous to clinical features) from a sparse true DAG with average graph degree 2–4. We then generated 2,500 variables (analogous to gene expression data *X*) based on the 50 latent factors (*Z*) and loadings (*A*) with weights drawn from a Gaussian distribution using Equation (2). To model the structure of regulation of gene expression, only 2% of the values of the combined loading matrix with the greatest absolute value were kept, and others were set to 0. This strategy controls the simulation so that each latent factor (e.g., TF) regulates approximately 50 gene expression features directly.


(2) $$\begin{array}{r} X=ZA+E \end{array}$$

*X* is the *n*×*p* gene expression data matrix, $Z\in R^{n\times K}$ denotes K latent variables for n samples, $A\in R^{p\times K}$ is the Transcription Factor Regulation Matrix assigning p variables to K groups. E~N(0,1) is an *n*×*p* matrix of independent error.

For the gene expression simulation, we randomly picked five 1,000-sample datasets (out of the 10,000 total simulated samples) from each of the LH and CG datasets. The reported results are averages across these 5 sub-datasets.

#### Benchmark methods

- **PCA** We consider Principal component analysis (PCA) as a comparable baseline method to EBMF, since it is one of the most popular dimensionality reduction methods. We pick the first K principal components of the observed features by the eigenvalue ratio test (42), where K is estimated by:

(3) $$\begin{array}{r} \hat{K}=\arg\underset{k\in \left\{1,2,\ldots ,\bar{K}\right\}}{\max}\frac{{\hat{\lambda }}_{k}}{{\hat{\lambda }}_{k+1}} \end{array}$$

where $\hat{\lambda_{1}},\hat{\lambda_{2}},\ldots$ are eigenvalues, and largest K cannot be greater than *min*(*n, p*)−1.

- **FCI** For causal structure in the presence of confounders, we use FCI, a popular approach that generates asymptotically correct results. Thus, we compare performance of FCI and our model on simulation datasets with 10 latent factors and 200 observed features.

#### Metrics

EBMF models were calculated using the flashr package available by the authors of (28). We then built the moralized undirected graph from the trained EBMF identified factors and the categorical features (clinical targets) using MGM with StEPS subsampling procedure (38) for optimal sparsity. The moralized graph is used as the initial graph for the PC−Max algorithm with StARS (43), which produced a final stable causal graph on which we evaluated the original graph recovery. The causal discovery algorithms were performed using the rCausalMGM package.

To evaluate the recovery of latent factors by EBMF, we computed the mean correlation coefficient (MCC) between the known data-generating factors and the estimated latent factors learned through EBMF. To compute MCC, we calculated all pairs of correlation coefficients between source and recovered latent factors. Then, we permuted the correlation matrix to maximize diagonal sum through solving a linear sum assignment problem. Finally, we assigned each recovered latent factor to best-correlated source latent factor, and computed MCC based on the diagonal of the permuted correlation matrix.

To evaluate the true graph recovery, we computed adjacency precision (AP) & recall (AR) and arrowhead (causal orientation) precision (AHP) & recall (AHR). Adjacency precision and recall refer to the correctness of the edges in the graph estimated by the causal discovery algorithms regardless of the corresponding orientation(s). Arrowhead precision and recall are computed using the true positive, false positive, false negative, and true negative orientations defined in Table 1 (29). This score is computed only on edges that appear in both the data generating graph and the estimated graph. Thus, this arrowhead score always is accompanied by adjacency score to separate the information gained from the adjacencies vs. arrowheads. The main idea of the score is to treat endpoint “>” as positive and endpoint “−” as negative. If the true graph contains edge A → B, and the estimated graph contains edge A → B, this counts as one true positive in A and one true negative in B. The precision and recall of adjacency and arrowhead are defined in Equation (4).


(4) $$\begin{array}{r} \begin{array}{c} \text{Adj Precision}=\frac{\text{correctly predicted adjacencies}}{\text{predicted adjacencies}}\\ \text{Adj Recall}=\frac{\text{correctly predicted adjacencies}}{\text{true adjacencies}}\\ \text{Arr Precision}=\frac{TP}{TP+FP}\;\text{Arr Recall}=\frac{TP}{TP+FN} \end{array} \end{array}$$

**Table 1:** Causal orientation score.

**Edge**	True: A*−>*B*	True: A*−−*B*
Predicted: A*−>*B*	TP	FP
Predicted: A*−−*B*	FN	TN

### Application to biomedical datasets

In order to test how well our high-dimensional CausalMGM performs in real-world biological applications, we applied the strategy to two clinically important gene expression datasets: (1) breast cancer and (2) SARS-CoV-2. For breast cancer, we use METABRIC (44), a microarray dataset with 1307 patients and 60 clinical features. For COVID-19, we use a recently published open-access RNAseq dataset (45) with 100 COVID-19 and 26 non-COVID-19 patients and 9 clinical features.

For both datasets, we used EBMF as the first step because the results on the simulated data showed that it outperformed PCA. We trained a greedy-backfitted EBMF model over all patient gene expression data to build the latent factor model. Then, we merged the known clinical features with the latent factors to train a causal network (using PC and FCI).

By definition, in a Bayesian network, all the information regarding a target is contained within its Markov Blanket. We used this concept to select important factors of target variables for further analysis.

The Mann-Whitney *U*-test was used to identify significant EBMF factors between groups. For Gene Set Enrichment Analysis (GSEA) (46) of factor loadings, the R function gseGO from the clusterProfiler package (47) was applied to the rank order of the EBMF factor loadings to identify Gene Ontology Biological Processes (48) enriched in a given factor loading. Enrichment dot plots for GSEA results were generated with the enrichplot package (49) in R.

For the METABRIC dataset, Kaplan-Meier estimates (50) and Cox Proportional Hazards model (51) of the disease-free survival curves are computed with the survival package (52). Optimal cutoff values for each factor in relation to disease-free survival were determined by a maximally selected rank statistic. The Kaplan-Meier estimates were then plotted with the survminer package (53).

For the SARS-CoV-2 dataset, the performance of predictive models for disease state and ICU admission were assessed by the area under the receiver operator characteristic (ROC) curve (AUC). These ROC curves were computed and plotted with the pROC package (54) using five-fold nested cross-validation. A baseline predictive model, constructed directly on gene expression data, was learned using logistic regression with elastic net regularization implemented in glmnet (55). Predictive models based on EBMF factor causal models were constructed by performing logistic regression on the Markov blanket of the target outcomes.

## Results

### Performance on synthetic data

#### Evaluation of latent factor recovery

**Figure 2:**
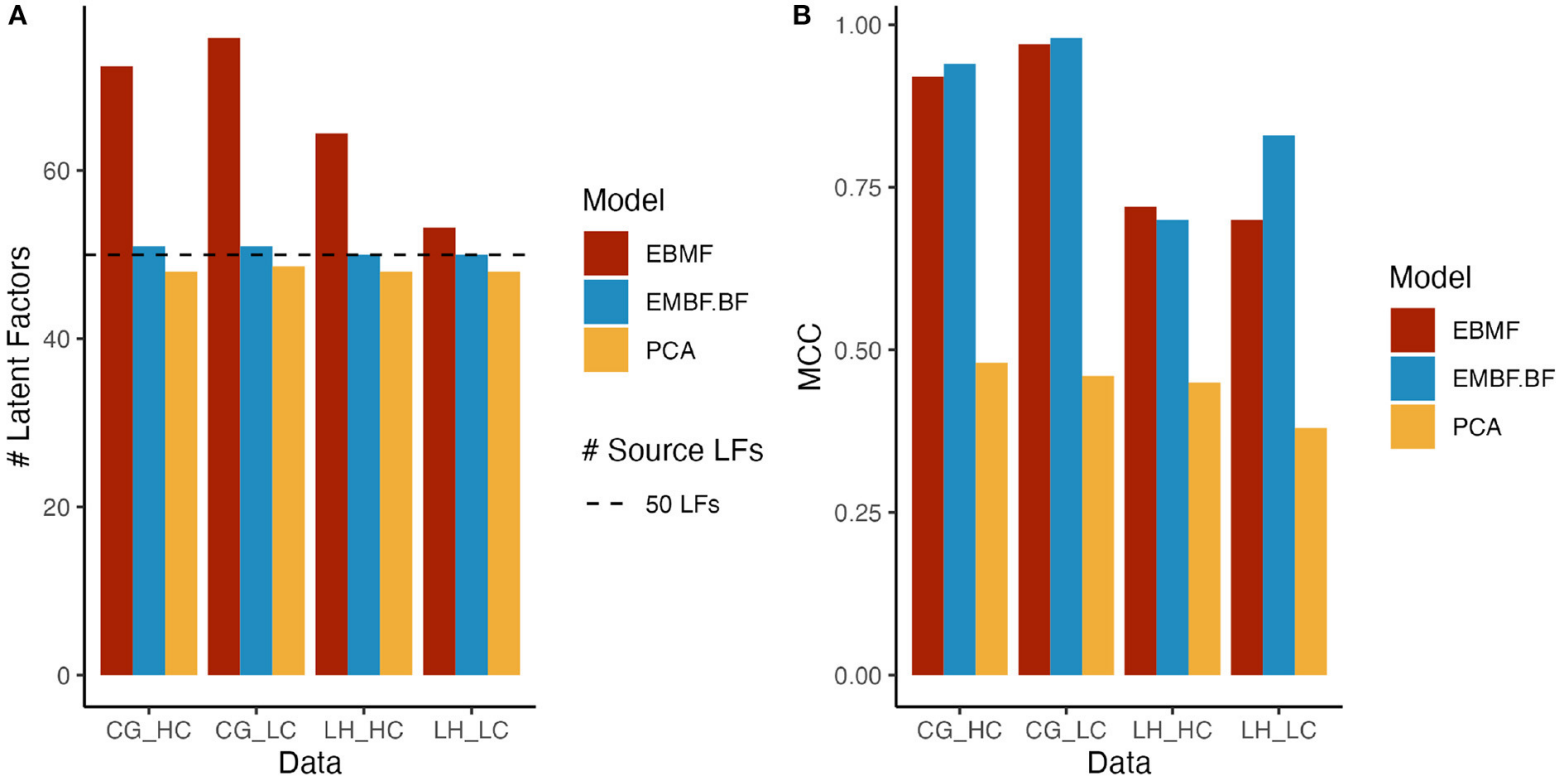
Bar plots for **(A)** number of latent factors and **(B)** mean correlation coefficient (MCC); EBMF represents latent factors recovered by greedy algorithm. EBMF+BF represents latent factors recovered by greedy algorithm + Backfitting algorithm; PCA represent principal component analysis; HC/LC represents simulated data with high/low correlation; the line at 50 latent factors indicates the ground truth number of source latent factors.

To evaluate the ability of EBMF to recover the true latent factors in a dataset, we applied both the greedy-only and greedy + backfitting algorithms on synthetic datasets. As a baseline, we also applied PCA with the number of principal components selected by the eigenvalue ratio test. We consider latent factor recovery to be successful if the method correctly identifies the number of latent factors, and the MCC of the recovered factors and true data generating factors is high. The greedy + backfitting algorithm and PCA with the eigenvalue ratio test perform well in selecting the correct number of latent factors (Figure 2A). However, the MCC of the learned latent factors and true data generating factors is much higher for EBMF compared to PCA (Figure 2B) across all simulation conditions, demonstrating that EBMF can better recover the source factors when compared to PCA. This is a consequence of the orthogonality constraint in PCA, which hinders the recovery of interacting, dependent latent factors. EBMF does not have this constraint, and can more precisely recover the true source latent factors for CG simulated data (Figure 2 and Supplementary Figure 1B). While the MCC for the LH simulated data is relatively lower (Figure 2 and Supplementary Figure 2B), indicating lower true factor recovery, it still outperforms PCA by a large margin.

#### Evaluation of causal graph recovery

Next, we performed MGM with StEPS on the EBMF greedy + backfitting discovered latent factors to identify optimal lambda values for an undirected graph. Using the undirected network generated by MGM, we ran PC−Max with StARS to determine an optimal alpha value for a stable graph. Table 2 summarizes the adjacency and arrowhead precision and recall, and the results are split by edge types: CC are edges between continuous variables, DD are edges between discrete variables, and CD are edges between mixed (continuous and discrete) variables.

**Table 2:** Graph recovery of simulated data (MGM-PC-MAX).

	**EDGE**	**CC**				**CD**			
**Data**	**TYPE**	**AP**	**AR**	**AHP**	**AHR**	**AP**	**AR**	**AHP**	**AHR**
CG-LC	Source	1.00	0.60	0.67	0.33	0.98	0.26	0.82	0.47
	EBMF+BF	0.86	0.14	0.65	0.18	0.98	0.29	0.83	0.18
CG-HC	Source	0.99	0.81	0.91	0.65	1.00	0.52	0.82	0.45
	EBMF+BF	0.74	0.10	0.83	0.27	0.89	0.52	0.50	0.20
LH-LC	Source	1.00	0.95	0.82	0.80	1.00	0.81	0.66	0.60
	EBMF+BF	0.65	0.24	0.64	0.51	0.74	0.67	0.64	0.54
LH-HC	Source	1.00	0.97	0.94	0.56	0.98	0.91	0.81	0.62
	EBMF+BF	0.19	0.03	1.00	0.50	0.53	0.85	0.62	0.56

Since we model the latent factors as continuous variables, we investigate the ability of our method to recover CC and CD edges. As a benchmark, we compare the performance of MGM-PC-Max applied to the EBMF recovered latent factors to MGM-PC-Max applied to the true, data generating source latent factors. This benchmark represents the best case causal discovery performance, in the scenario where the latent factors are perfectly reconstructed. On Conditional Gaussian simulated data, causal discovery on EBMF estimated latent factors performs similarly well to casual discovery on the source factors in terms of recovering interactions between the continuous latent factors and the categorical “clinical” features. EBMF struggled to learn interactions between latent factors, especially in the high correlation setting, but reasonably high adjacency precision indicate that most edges that are inferred by MGM-PC-Max on EBMF latent factors are true causal interactions. However, MGM−PC−Max has worse performance on EBMF recovered latent factors from Lee and Hastie simulated data. This is especially true for the high correlation case (Figure 2B). This difficulty with recovering latent factors in Lee & Hastie simulated data results in a failure to identify true causal interactions within the latent factors (Table 2). This performance difference is due to the higher internal covariance between continuous variables and discrete variables in Lee & Hastie model data, making it more difficult to recover the true source latent factors. This indicates that strong associations between continuous and discrete variables can cause EBMF to struggle with identifying the true latent factors. However, when compared to PCA and FCI, EBMF was able to recovery of true latent factors at a much higher accuracy, which is critical for the subsequent causal discovery.

We also compare our approach with a baseline method, FCI, on smaller simulation datasets with 10 latent factors and 200 observed features. While FCI is not designed to recover the actual latent factors, it can infer whether two variables are confounded. We inferred the expected confounded edge adjacency matrix from the loading matrix (for source loading and EBMF loading), and we compared this to the confounding edges inferred by FCI. This allows us to compute adjacency recall & precision for confounded edges from the FCI output PAG. For PAG, We count one double-arrow edge (↔), which indicates that two variables are confounded, as one True Positive (TP); one double circle edge (o-o), which indicates confounding but also possible direct cause-effect in either direction, as one third TP and two thirds False Positive (FP); and one *o* → edge, which indicates confounding or possible cause-effect in one direction, as one half TP and one half FP. Overall, EBMF has a higher recall and relatively high precision. In comparison, FCI has a much lower recall and relatively lower precision (Table 3). This demonstrates that our model, in addition to recovering the values of the latent factors and feature loadings themselves, can identify confounded features more efficiently than FCI.

**Table 3:** Adjacency recovery of loading matrix.

**Data**	**EBMF-AP**	**EBMF-AR**	**FCI-AP**	**FCI-AR**
CG_LC	1	1	0.53	0.19
CG_HC	0.67	0.98	0.51	0.18
LH_LC	0.63	0.99	0.53	0.21
LH_HC	0.44	0.99	0.51	0.20

### Performance on biomedical disease data

#### Application to METABRIC breast cancer dataset

After removing features and patients with missing data, the METABRIC dataset contained 1,221 patients with 17,268 genes and 14 clinical features. The clinical features included can be seen in Supplementary Table 1. From the 17,268 gene expression features, the greedy + backfit EBMF identified 328 latent factors. The resulting latent factors also have low pairwise correlations, indicating that EBMF has successfully reduced the multicollinearity of the dataset (Supplementary Figure 3).

**Figure 3:**
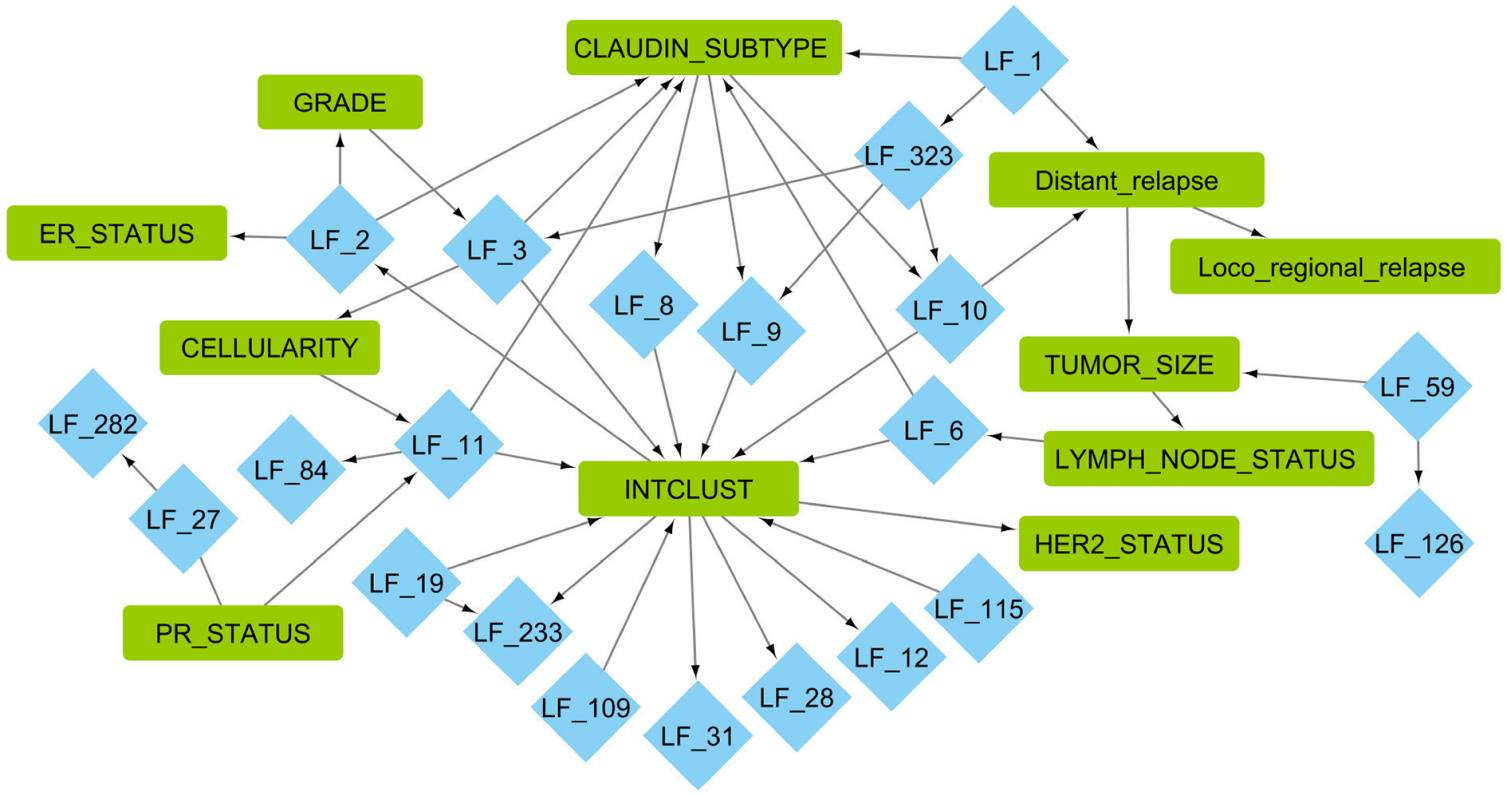
Markov blankets of important clinical features from the causal network learned using MGM−PC−Max. Blue diamonds are factors learned from METABRIC gene expression using greedy backfitted EBMF. Green squares are clinical METABRIC features. For explanation of the clinical features, please see Supplementary Table 1.

Using these 328 EBMF factors and 14 clinical features, we ran MGM to build an undirected skeleton graph, which was used as the initial graph for PC−Max. The StARS subsampling procedure yielded the most stable graph that connected the latent factors and clinical features. A selected subset (first and second neighbors of clinical features) of the network can be seen in Figure 3.

**Figure 4:**
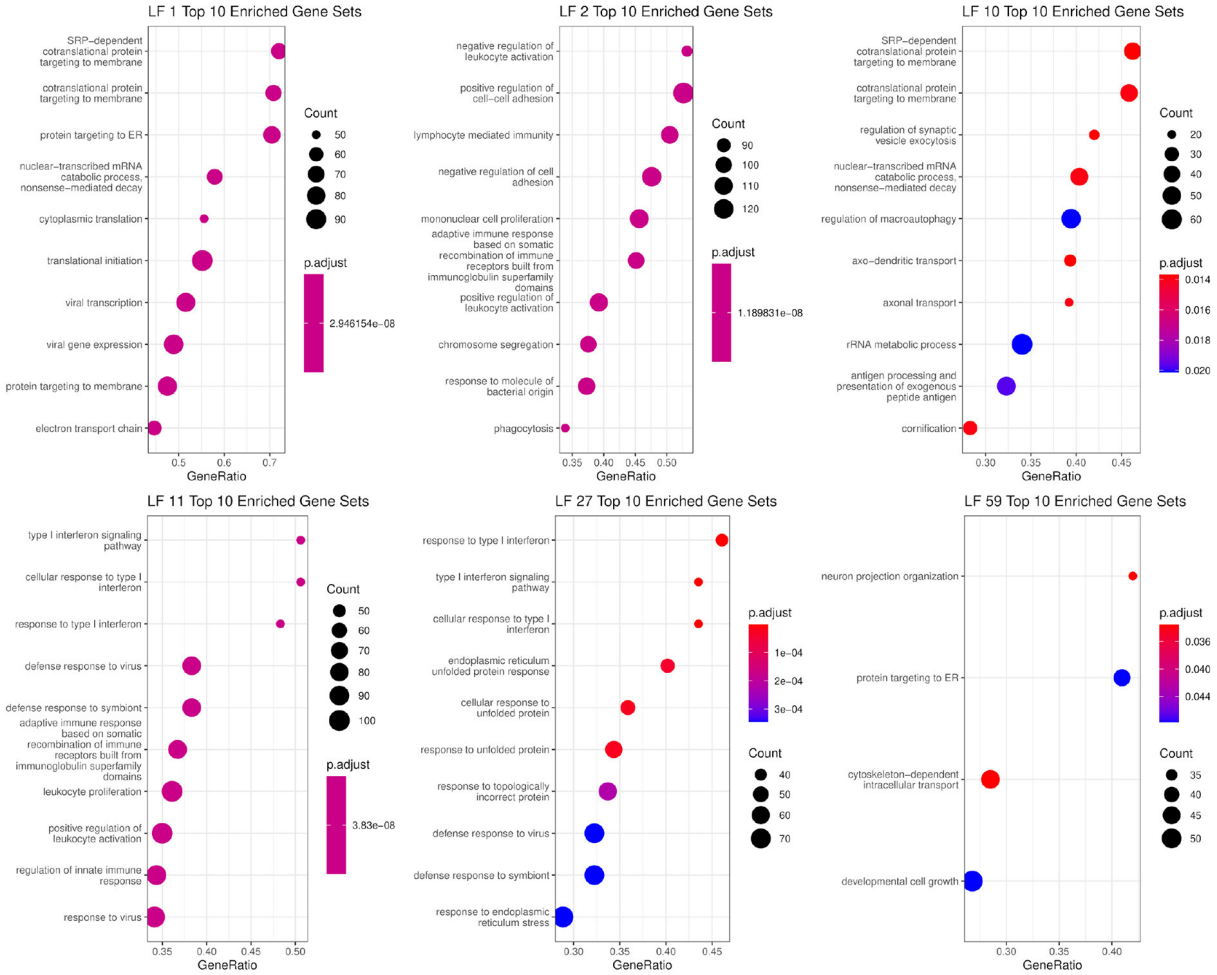
Biological functions significantly associated with factors that are in the Markov blankets of tumor size: LF59, distant relapse: LF1 and LF10, ER status: LF2, PR status: LF11 and LF27.

We then evaluated this graph by examining the EBMF factors that are in the Markov blanket of the clinical features. The associated factors were used for gene set enrichment analysis in order to identify the enriched biological processes. The top 10 gene sets with ratios, adjusted *p*-value, and total weight can be seen in Figure 4. Some factors had less than 10 processes associated with them with FDR adjusted *p* < 0.05, such as LF59.

In the Markov blanket of ER status, we see one factor, LF2, which has positive values for ER+ patients, and negative values for ER- patients (*p*-value < 2.2e-16, Supplementary Figure 4). LF2 is associated with immune response, interaction, and signaling pathways, which is matches existing literature about how ER activity can regulate immune signaling pathways and cytokine production (56). Previous literature has also demonstrated that the majority ER+ patients have lower levels of tumor infiltrating lymphocytes (57) compared to triple-negative patients.

Two factors (LF11 and LF27) are directly linked to PR status, and both are associated with immune response. In particular, the genes comprising these factors belong to interferon signaling pathways based on GSEA analysis. Those genes are also associated with viral immune response. Recent work has shown that PR+ tumors exhibit lower levels of phospho-STAT1, which in turn attenuates interferon-induced STAT1 signaling, which in turn may allow PR+ tumors to escape immune surveillance (58).

In the Markov blanket of tumor size, there is one factor LF59 as well as distant relapse and lymph node status. LF59 is associated with protein targeting to the endoplasmic reticulum. Endoplasmic reticulum stress is known to be directly linked to tumor growth inhibition and apoptosis (59, 60). It is also associated with cell growth and structure related gene sets, which are needed for tumor cell growth.

For distant relapse, we see factors LF1 and LF10. LF1 and LF10 are positive for survivors, and negative for early, middle, and late recurrence patients. LF1 is associated with RNA translation, processing, and quality control pathways. There is also an association with the biological pathways associated with viral immune system response. LF10 is associated with pathways connected RNA processing and quality control, cell death promoters, and translation initiation, elongation, and termination. In general, aberrant RNA quality control and protein translation plays an important role in cancer pathogenesis (61).

**Figure 5:**
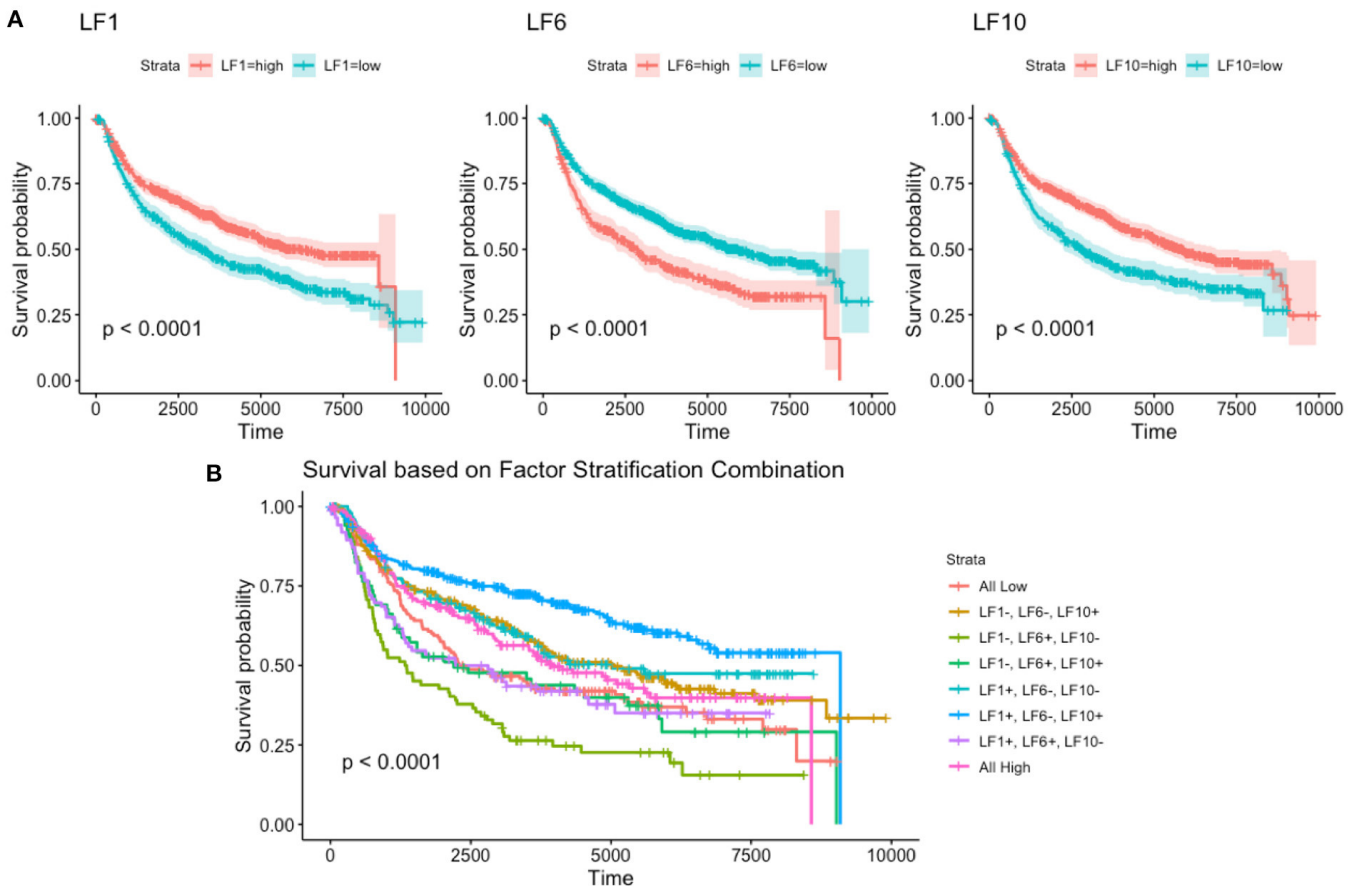
Disease free survival based on **(A)** individual factors and the **(B)** combinations of all factors.

For each of these four continuous factors, we were able to determine the optimal cut-point to split the survival into high and low categories (53). Using these cutoffs, we were then able to build disease-free survival curves for each of the factors (Figure 5). We trained a multivariate Cox Proportional Hazards model using these factors to determine their contribution to disease-free survival (Supplementary Table 2). From the figure, we see that factors LF1, LF6, and LF10 have significant *p*-values, while LF59 does not. We exclude LF59 from further analysis for this reason. Using the significant factors LF1, LF6, and LF10, and their respective cutoffs, we are able to generate eight survival curves to represent the eight possible high/low combinations of the factors (Figure 5). These eight groups are able to stratify patients into significantly different disease-free survival curves, demonstrating that the recovered latent factors recover biologically relevant information about a key clinical outcome.

#### Application SARS-CoV-2 dataset

**Figure 6:**
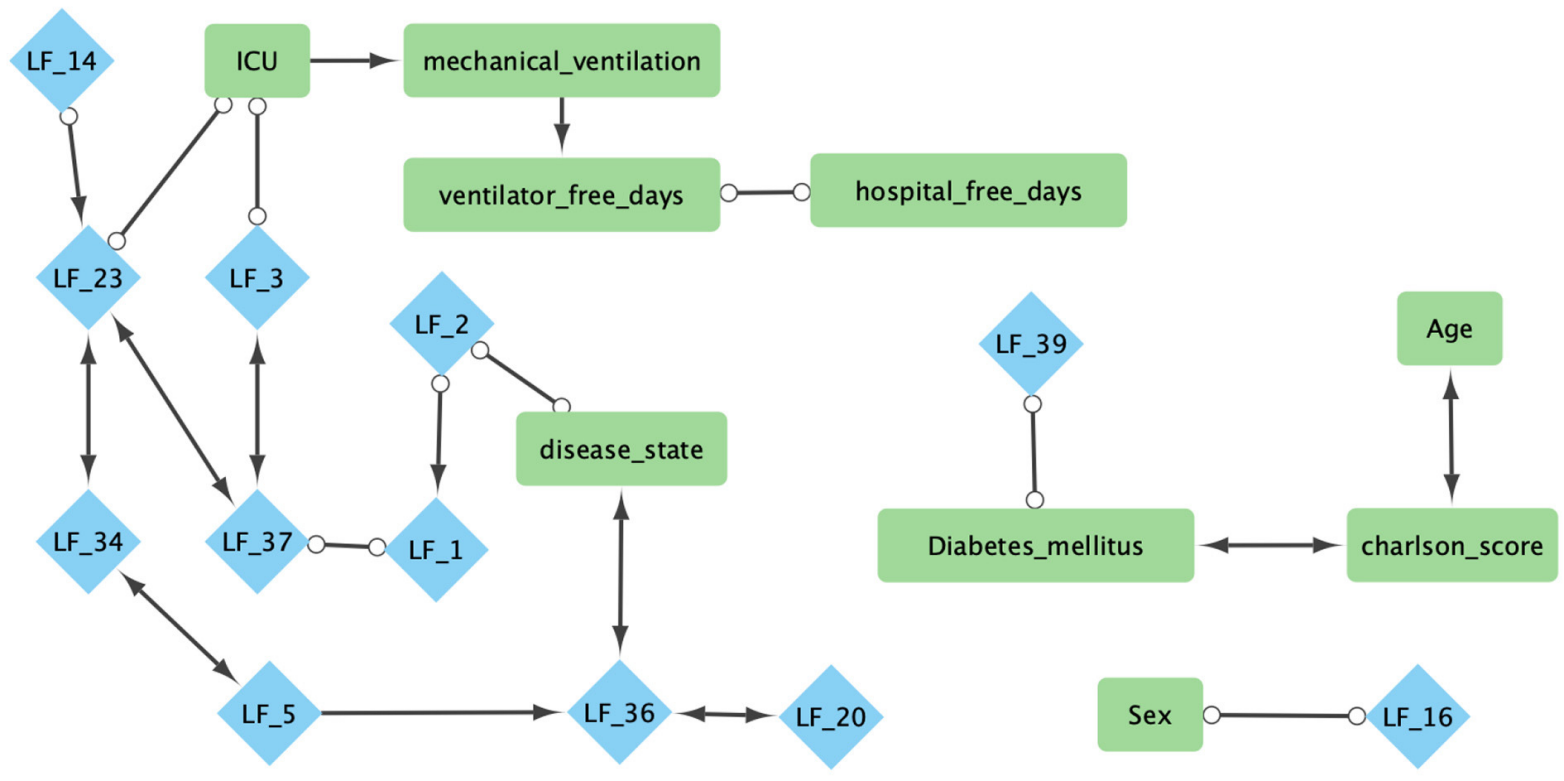
Combined Markov blankets of clinical features from causal network learned using FCI−Max with bootstrapping, highest ensemble, and α = 0.05. Blue diamonds are factors learned from gene expression using greedy-backfitted EBMF. Green squares are clinical features. For explanation of the clinical features, please see Supplementary Table 3.

In the COVID-19 RNA-seq dataset (19,472 genes), the backfitted EBMF identified 40 latent factors. Using FCI with bootstrapping and an α = 0.05, we learned a stable (edge appearance >50%) causal network from the latent factors and clinical variables. The majority of the network (26/30 features, 28/32 edges) was within the Markov Blanket of the clinical features, and can be seen in Figure 6. Two features of particular interest are disease state and ICU admittance (which we used as a proxy for disease severity). The Markov Blanket of disease state contains factors LF2, LF5, LF20, and LF36. The Markov Blanket of ICU admittance contains LF3 and LF23.

**Figure 7:**
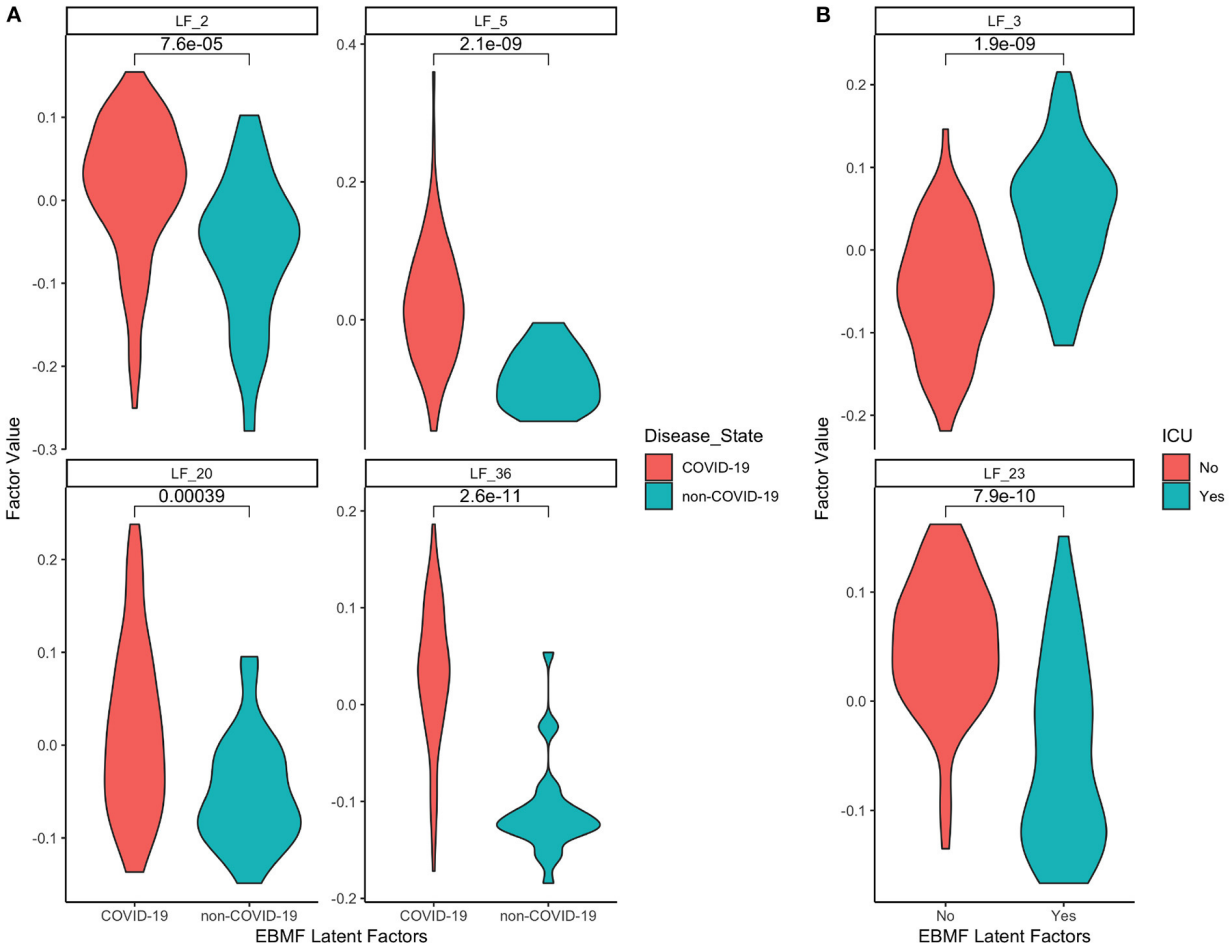
Distributions of EBMF factor values for features in the Markov blanket of **(A)** disease state (COVID-19 vs. non-COVID-19), **(B)** ICU admittance (yes vs. no).

Next, since the dataset contained both COVID and non-COVID patients, we were able to split these estimated EBMF factor values (LF2, LF5, LF20, LF36) based on patient disease class. This allows us to compare the distribution of values between these two groups. The results can be seen in the violin plots in Figure 7A. In the plots, we can see that the average derived value for COVID-19 patients in all four latent factors is positive, while the average derived value for non-COVID-19 negative, with significant *p*-values distinguishing the two groups. This indicates that the causal algorithm is finding factors directly linked to whether the patient has COVID-19. Additionally, this also means that genes that are positively linked to LF2 are overexpressed for COVID-19 patients, while genes that are negatively linked to LF2 are overexpressed in non-COVID-19 patients.

The same strategy can be used for patient ICU admittance (LF3, LF23) since it is also a binary category. The relative distributions of those who had to enter the ICU vs. those who didn't can be seen in Figure 7B. We see that patients who enter the ICU have positive average factor values for LF3, and negative average factor values for LF23 (and vice versa for those who don't enter the ICU). This indicates that genes positively associated with LF3 will be positively linked to ICU admittance, while genes positively associated with LF23 will be negatively linked to ICU admittance.

**Figure 8:**
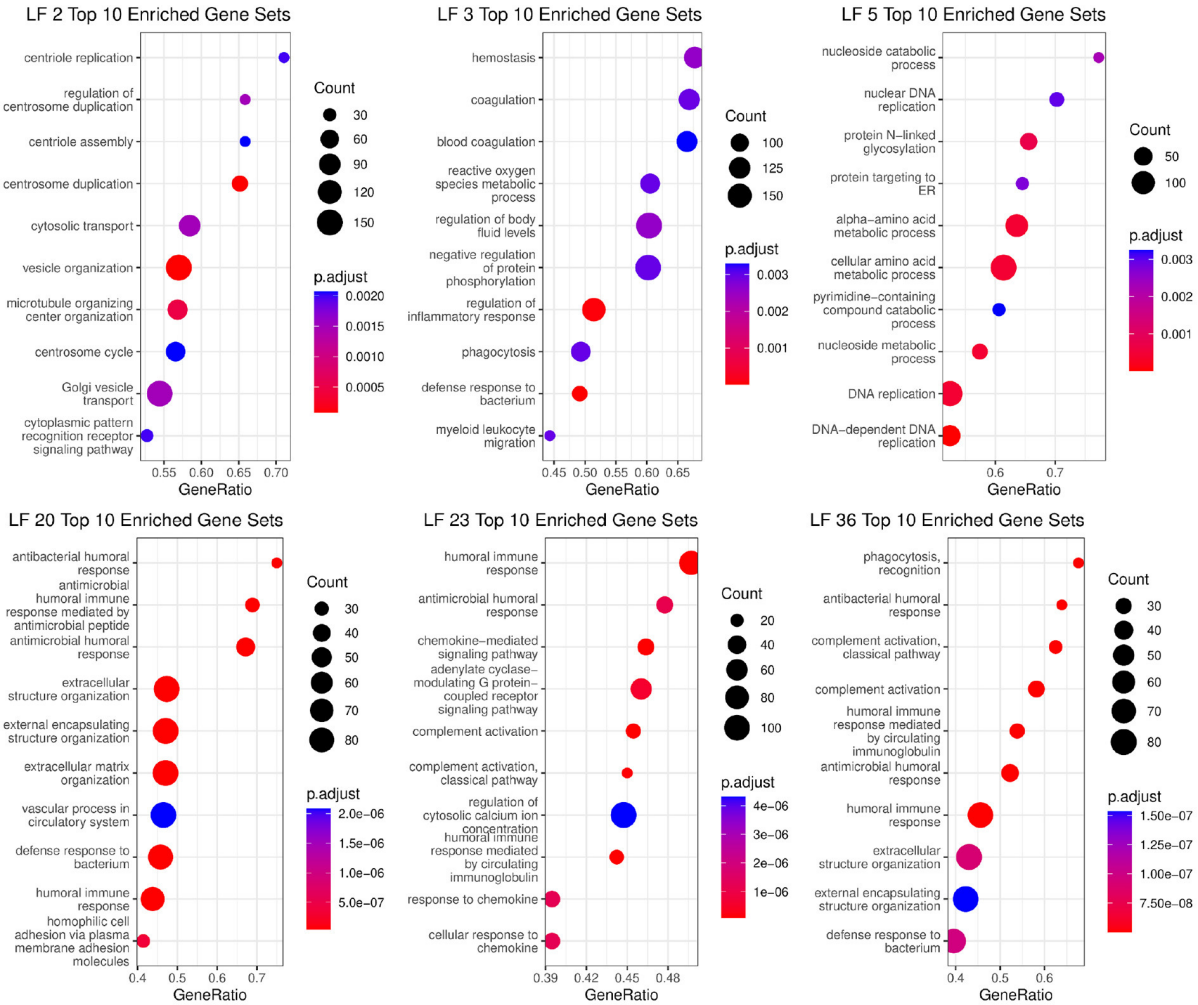
Biological functions significantly associated with Factors that are in the Markov blanket of disease state (LF2, LF5, LF20, LF36) and ICU Admittance (LF3, LF23).

Using pathway enrichment analysis on the loading gene weights that made up each factor, we were able to identify the biological mechanisms that each factor was associated with. The gene sets with ratios, adjusted *p*-value, and overall total weight can be seen in Figure 8. In the figure, we see that the factors (LF2, LF5, LF20, LF36) associated with COVID-19 disease state have high enrichment for innate & adaptive immune response, metabolism and autophagy, which matches expected response to viral infections and is supported by COVID-19 literature (62). For ICU admittance, we see enrichment in crucial pathways that are known to be closely associated with COVID-19 severity, such platelet activation and coagulation (63), and chemokine activity (64). We also see many other immune response pathways within the top 10 pathways within all 6 factors.

**Figure 9:**
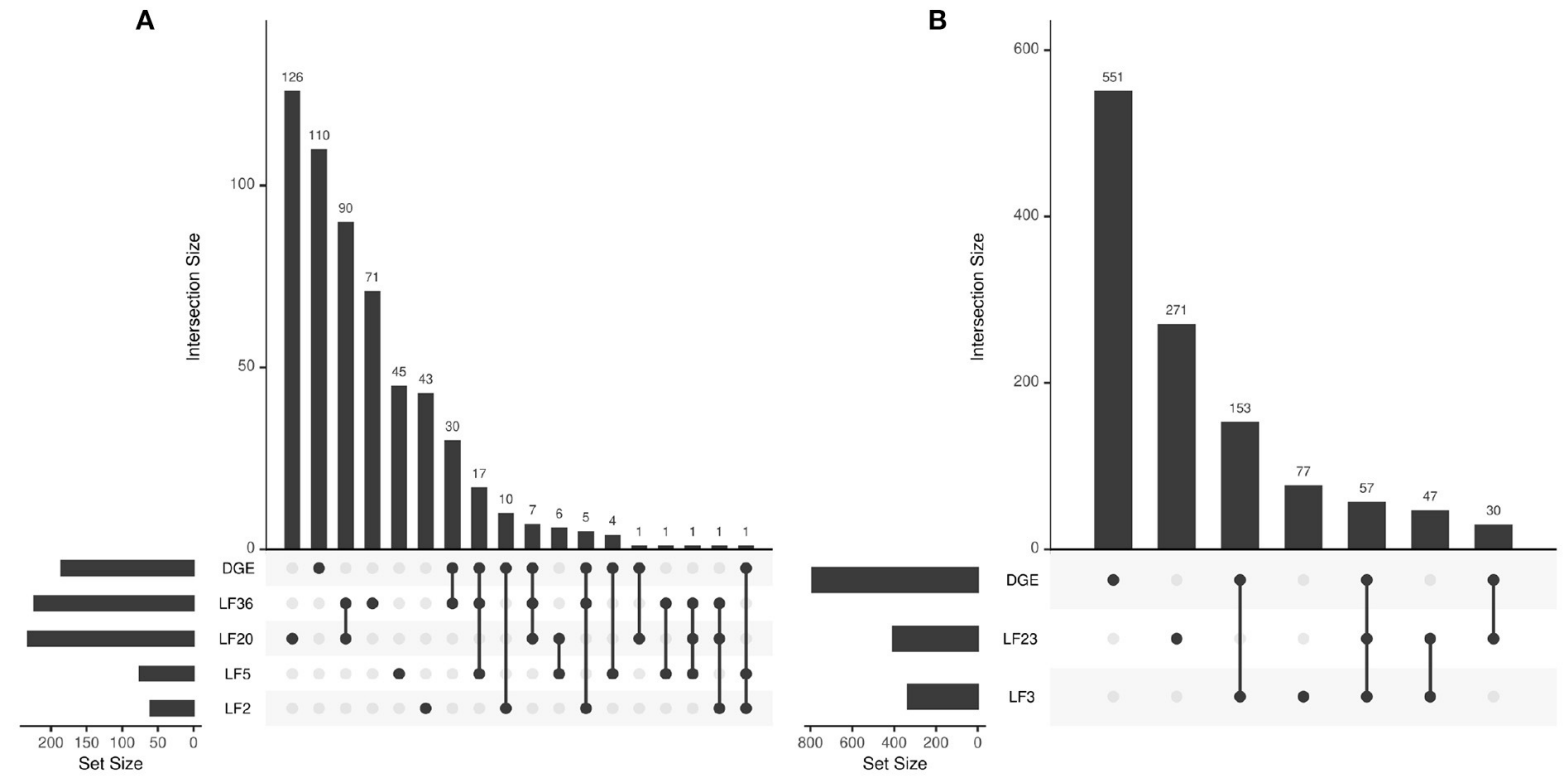
Intersection between biological function gene sets that are significantly associated with the differentially expressed genes and LFs across **(A)** disease state and **(B)** ICU admittance.

We also compare the individual factors to the list of biological functions significantly associated with the complete differentially expressed genes (DGEs) result. The UpSet plots in Figure 9 describe the overlap between the DGE gene sets and the factors' gene sets. We can see individual factors capture subsets of the main DGE gene sets. For example, there are 62 significant (p < 0.05 in both) gene sets that are overlapping between the main DGE and LF36. Similarly, we see 210 significant overlapping gene sets between ICU DGE and LF3. The results also demonstrate that the factors contain novel information that the full dataset DGE results do not contain. For example, LF36 has 71 unique significant gene sets that are not shared with any other COVID-19 disease state associated factors or the complete DGE analysis. This indicates that the factors are capturing both general biological information about COVID-19 disease state and ICU admittance that is contained in the overall dataset, but is also able to capture detailed differences that full dataset analysis would otherwise miss.

**Figure 10:**
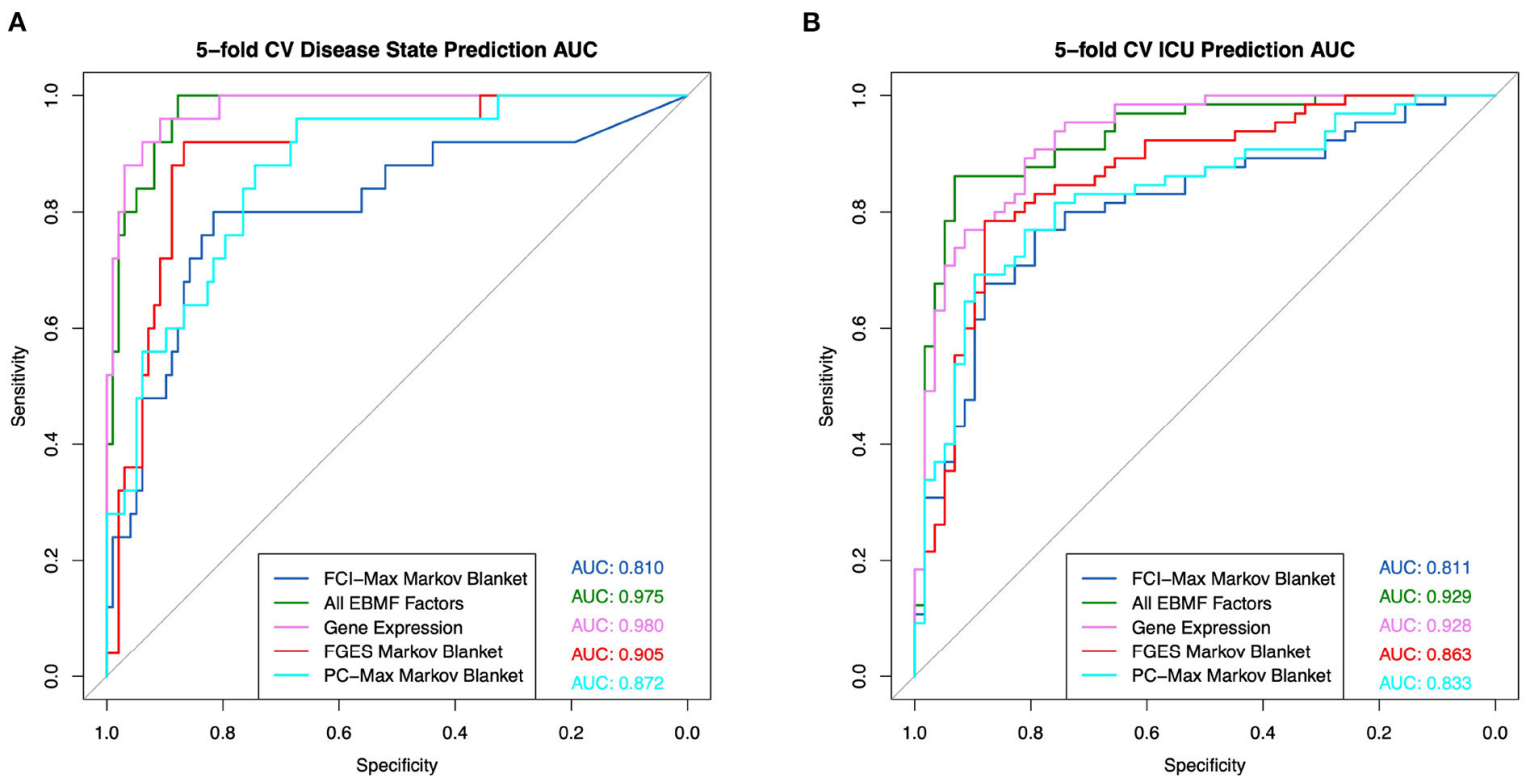
General linear model prediction ROC using factors contained within the Markov blanket for **(A)** disease state and **(B)** ICU admittance.

The Markov Blanket of a clinical feature can also be used for feature selection for clinical prediction (9). Using five-fold nested cross validation, we were able to build general linear models to predict whether a patient had COVID, and whether a patient would enter the ICU. Note that, in order to avoid overfitting, the entire causal learning process + Markov Blanket feature selection needs to be done for each fold (nested cross-validation). The ROC plots are presented in Figure 10. These results show that classifiers based on the EBMF factors are equally good as those using all differentially expressed genes.

## Discussion

In this paper, we have proposed a two-step system to build causal models from high-dimensional multicollinear datasets. In step-1, we use EBMF to derive latent factors (e.g., TFs) from the observed variables (e.g., Gene Expression). In step-2, we perform causal discovery on the latent factors and clinical features (e.g., Age, Response).

In simulation studies we show that both EBMF and PCA can identify an accurate number of latent factors. However, only the EBMF recovered latent factors are highly correlated with the (known) source latent factors. Additionally, the latent factors learned by EBMF are not strictly orthogonal, which enables them to be used for causal discovery. We also demonstrate that our method can recover causal interactions between latent factors and clinical covariates with a high degree of accuracy.

Application to breast cancer and SARS-CoV-2 gene expression data shows that EBMF is able to significantly reduce the dimensionality of these datasets. It is also able to reduce to the multicollinearity within the datasets. The factors in the Markov blanket of clinical features also contained significant biologically relevant pathway information that is supported by existing literature.

For breast cancer, factors related to ER and PR status are associated with known immune signaling pathways and immune cell activity. Tumor size is associated with cell growth and tumor growth inhibition protein targeting pathways. Distant relapse linked factors contain important RNA and protein quality control pathways. Using the aforementioned factors, we were able to stratify breast cancer patients into distinct survival groups, where the best prognosis groups having over 50% survival rate at 7,500 days, and the worst having less than 25% survival rate.

For SARS-CoV-2, there is clear significant difference between the COVID-19 and non-COVID-19 patient distributions for the four latent factors related to disease state. These factors contain immune pathway genes that match known viral immune responses. Similarly, there is a significant difference in factor value distributions between ICU and non-ICU patients for the two important related factors. These ICU factors matched known biological pathways associated with severe immune response.

We also used EBMF with causal discovery as feature selection method in order to do clinical prediction of patients with COVID-19, and the severity of the patient's case. We were able to predict with high accuracy both whether a patient has COVID-19 and whether they will enter the ICU. These results were achieved with linear models, which cannot fully capture the non-linear relationships that exist in biological datasets. Therefore, the accuracy could increase even further with non-parametric models (e.g., random forests). Interestingly, we were also able to find factors associated solely with sex and with diabetes status, which may be interesting starting points for future research.

Due to our structural assumptions, the performance of our framework may suffer if there are many interactions among observed features (RNA-RNA interactions). RNA-RNA interactions typically include non-coding RNA (ncRNA)-ncRNA or ncRNA-message RNA (mRNA). Direct interactions between mRNA-mRNA are rare because they usually have to go through protein products and post-translational signaling. Research shows that stable mRNA-mRNA interactions are sometimes detected *in vivo* (65), but very few mRNA-mRNA interactions are involved in gene regulation, such as hly mRNA binding prsA2 mRNA and protect it from degradation (66, 67). The lack of regulatory function means that it will not have a large effect on the gene expression, so in gene expression datasets containing mRNA features only, mRNA-mRNA interactions can be ignored. When working with whole transcriptomics data, which can contain other RNA types (such as ncRNA), there may be more regulatory RNA-RNA interactions. In this case, we may overlook some possible dependencies, which is a limitation of our method.

The authors recognize that the model proposed is not a complete biological picture since it makes assumptions about the internal structure and relationships between features. However, based on our current understanding of gene expression regulation, we argue that it is a justified and reasonable approximation. Additionally, all causal discovery methods are at best approximations when it comes to analyzing gene expression data since the data are often averages of many cells (e.g., bulk RNAseq or microarray), have systematic bias (e.g., single cell RNAseq dropout), or have cyclic relationships, which violate (to some degree) the assumptions of most existing causal discovery algorithms. Despite these drawbacks, the success of probabilistic graphical models in analyzing biomedical data (68–70) has shown that they can still be good approximations. Our own simulation and application results demonstrate the potential of EBMF CausalMGM for gene expression data.

## Data availability statement

The simulated data and code can be found this github link (https://github.com/Lobbii/HD_CL_causal_discovery). The Covid19 dataset for this study can be found in the GSE157103. The breast cancer dataset for this study can be found in cbioportal (http://www.cbioportal.org/study/summary?id=brca_metabric). Further inquiries can be directed to corresponding authors.

## Author contributions

MJ: study design, performed simulation data analysis, interpreted simulation results, and wrote the paper. DYY: study design, performed biomedical data analysis, interpreted biomedical results, and wrote the paper. TCL: study design, performed biomedical data analysis, and helped with paper writing. MH: preliminary data analysis. PVB: study design, aided in interpreting the results, and edited the paper. All authors contributed to the article and approved the submitted version.

## Funding

This work was supported by the National Institutes of Health (R01 HL157879, R01 HL159805, R01 AA028436, R01 DK130294, R01 HL127349 [PVB], T32 CA082084 [DYY], F31 LM013966 [TCL].

## Conflict of interest

The authors declare that the research was conducted in the absence of any commercial or financial relationships that could be construed as a potential conflict of interest.

## Publisher's note

All claims expressed in this article are solely those of the authors and do not necessarily represent those of their affiliated organizations, or those of the publisher, the editors and the reviewers. Any product that may be evaluated in this article, or claim that may be made by its manufacturer, is not guaranteed or endorsed by the publisher.
