# Supplementary material for: Causal discovery in high-dimensional, multicollinear datasets
Source: Front Epidemiol. 2022 Sep 13;2:899655. doi: 10.3389/fepid.2022.899655 (PMC9910507; doi:10.3389/fepid.2022.899655)
Supplement: Supplementary file 1 [file Data_Sheet_1.PDF]

Supplementary Material

1 SUPPLEMENTARY TABLES AND FIGURES

1.1 Tables

Feature	Description
AGE_AT_DIAGNOSIS	Age of patient at cancer diagnosis (years)
CANCER_TYPE_DETAILED	Detailed cancer type: Breast Invasive Ductal Carcinoma, Breast Invasive Lobular Carcinoma, Breast Mixed Ductal and Lobular Carcinoma, Breast Invasive Mixed Mucinous Carcinoma
CELLULARITY	Percentage of tumor volume occupied by invasive tumors cells categorized as Low, Moderate, High
CLAUDIN_SUBTYPE	Molecular breast cancer subtype defined by gene expression patterns: Luminal-A, Luminal-B, HER2+, Claudin-Low, Basal
Distant_relapse	Time of post-surgery distant relapse (non-breast/regional lymph tissue). Categories: Early (within 2 years), Middle (2-5 years), Late(5+ years), Survivor (no relapse)
ER_STATUS	Estrogen receptor status (Positive/Negative)
GRADE	Histological grade (1, 2, 3)
HER2_STATUS	Human epidermal growth factor receptor 2 status (Positive/Negative)
INFERRED_MENOPAUSAL_STATE	Patient Menopausal State (Pre/Post)
INTCLUST	Integrative cluster molecular subtype defined on gene expression and copy number variation
Loco_regional_relapse	Time of post-surgery local relapse (breast/regional lymph nodes). Early (within 2 years), Middle (2-5 years), Late (5+ years), Survivor (no relapse)
LYMPH_NODE_STATUS	Number of lymph nodes where tumor cells are seen: 0, 1 to 3, 4 to X
PR_STATUS	Progesterone receptor status (Positive/Negative)
TUMOR_SIZE	Log2 size of tumor (cm)

Table S1. METABRIC Dataset Clinical Features

Factor	Hazard Ratio	p-value
LF1	0.88 [0.82, 0.94]	< 0.001
LF6	1.22 [1.12, 1.32]	< 0.001
LF10	0.84 [0.78, 0.91]	< 0.001
LF59	1.01 [0.93, 1.09]	0.81

Table S2. Cox Proportional Hazards Model Factor Hazard Ratio

Feature	Description
disease_state	Whether the patient had Covid19 (yes/no)
Age	Patient age (years)
Sex	Patient sex (male/female)
ICU	Whether the patient entered the ICU (yes/no)
charlson_score	Charlson comorbidity index score (0-11)
mechanical_ventilation	Whether the patient needed mechanical ventilation (yes/no)
ventilator_free_days	Number of ventilator free days over continuous 45-day period (0-28)
Diabetes_mellitus	Whether the patient has diabetes mellitus (yes/no)
hospital_free_days	Number of hospital free days over continuous 45-day period (0-44)

Table S3. Covid19 Dataset Clinical Features

1.2 Figures

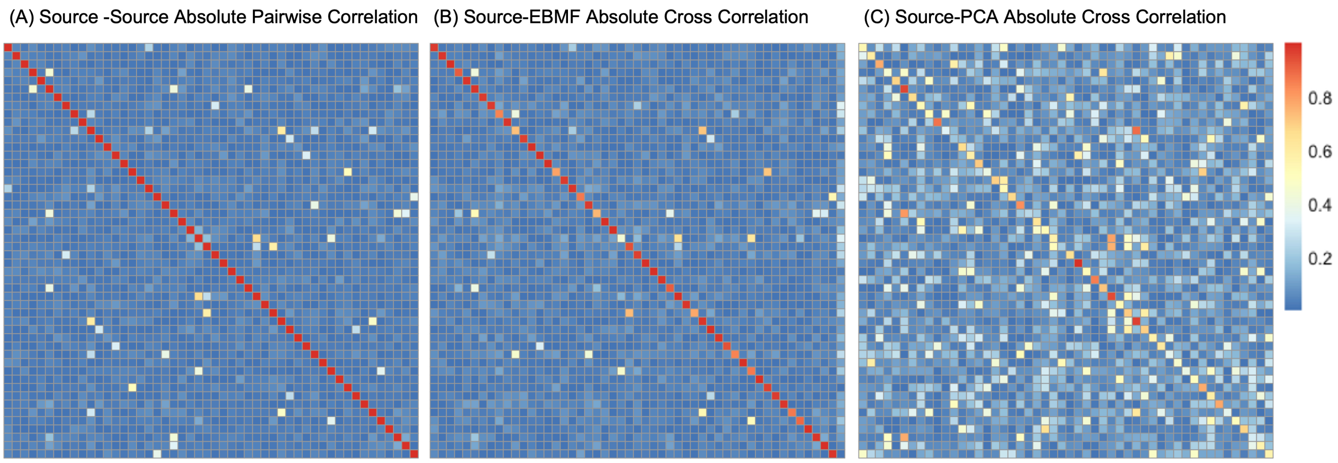

Figure S1. Absolute Correlation Heatmaps for Latent Factors of Conditional Gaussian Simulated Data with High Correlation (A) Source to Source (X- and Y-axis) (B) backfit-EBMF (X-axis) to Source (Y-axis) (C) PCA (X-axis) to Source (Y-axis).

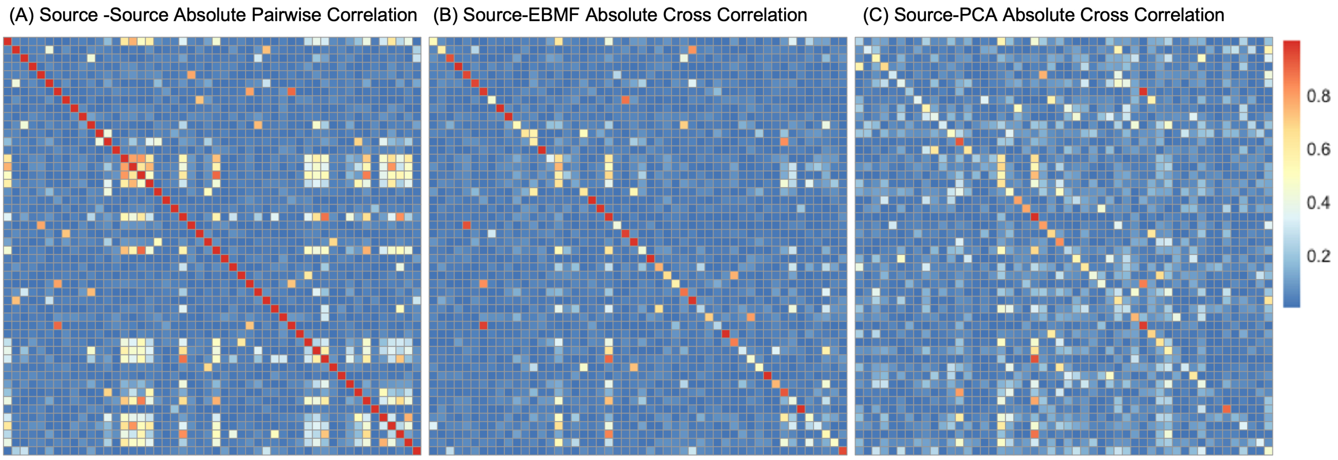

Figure S2. Absolute Correlation Heatmaps of Latent factor of Lee & Hastie Simulated Data with High Correlation: (A) Source to Source (X- and Y-axis) (B) backfit-EBMF (X-axis) to Source (Y-axis) (C) PCA (X-axis) to Source (Y-axis).

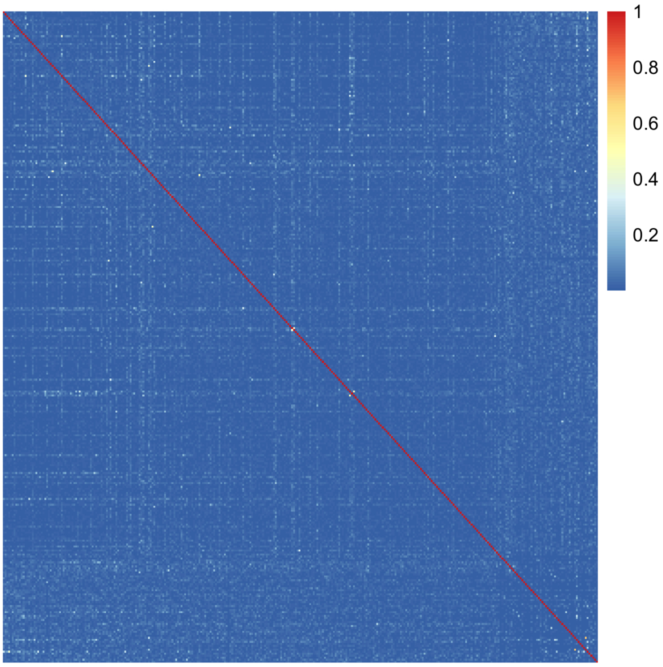

Figure S3. Absolute Pairwise Correlation Heatmaps of Latent factor of METABRIC Data.

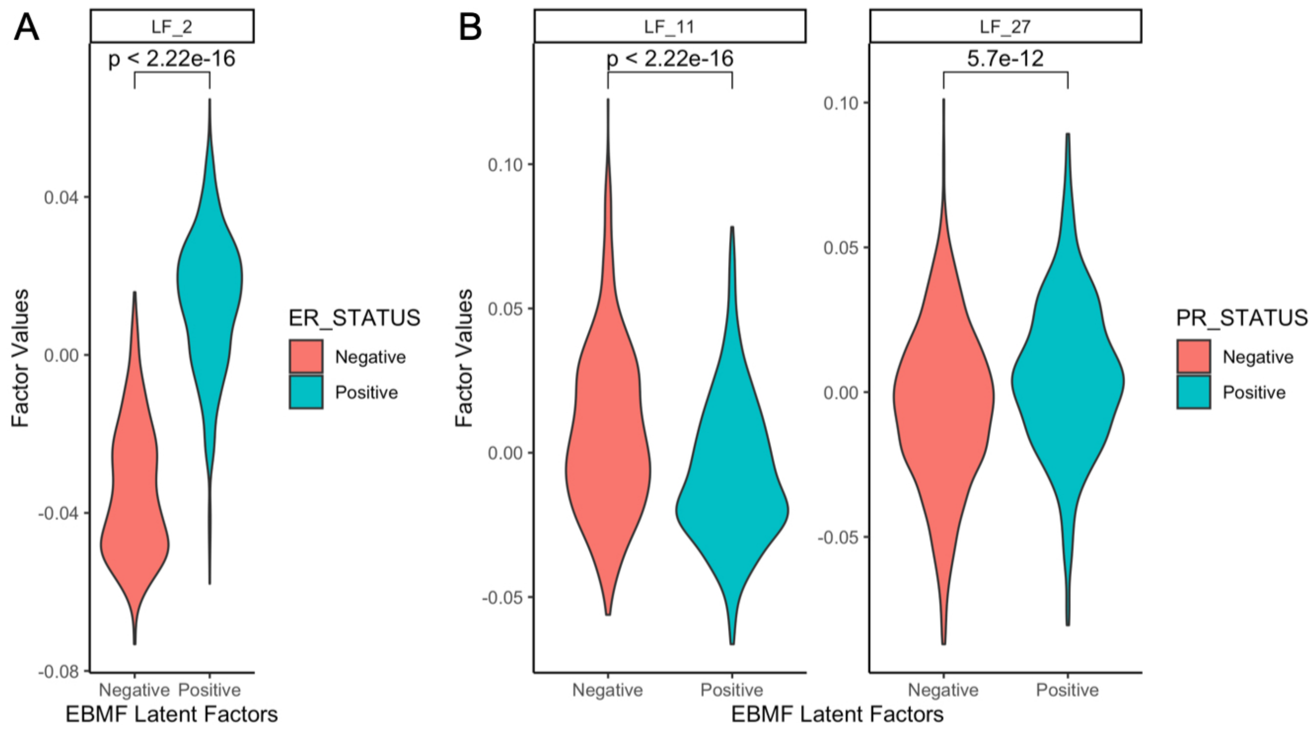

Figure S4. Distributions of EBMF Factor Values for features in the Markov blanket of (A) ER Status (B) PR Status

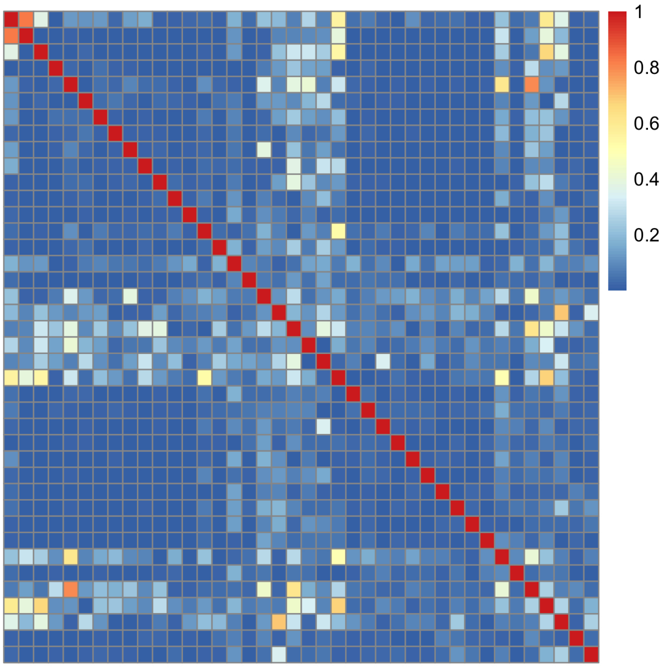

Figure S5. Absolute Pairwise Correlation Heatmaps of Latent factor of COVID-19 Data.
